# Multi-Stream Convolutional Neural Networks for Rotating Machinery Fault Diagnosis under Noise and Trend Items

**DOI:** 10.3390/s22072720

**Published:** 2022-04-01

**Authors:** Han Dong, Jiping Lu, Yafeng Han

**Affiliations:** 1 School of Mechanical Engineering, Beijing Institute of Technology, Beijing 100081, China; 3120190313@bit.edu.cn; 2 Changjiang Delta Institute, Beijing Institute of Technology, Jiaxing 314001, China; jipingLu@bit.edu.cn

**Keywords:** fault diagnosis, convolutional neural network, interfering signal, information fusion

## Abstract

In recent years, rotating machinery fault diagnosis methods based on convolutional neural network have achieved much success. However, in real industrial environments, interfering signals are unavoidable, which may reduce the accuracy of fault diagnosis seriously. Most of the current fault diagnosis methods are of single input type, which may lead to the information contained in the vibration signal not being fully utilized. In this study, theoretical analysis and comprehensive comparative experiments are completed to investigate the time domain input, frequency domain input, and two types of time–frequency domain input. Based on this, a new fault diagnosis model, named multi-stream convolutional neural network, is developed. The model takes the time domain, frequency domain, and time–frequency domain images as input, and it automatically fuses the information contained in different inputs. The proposed model is tested based on three public datasets. The experimental results suggested that the model achieved pretty high accuracy under noise and trend items without the help of signal separation algorithms. In addition, the positive implications of multiple inputs and information fusion are analyzed through the visualization of learned features.

## 1. Introduction

Rotating machinery, as key mechanical devices, is ubiquitous in modern industry. In engineering practice, rotating machinery frequently serves in harsh and complex environment with high speed, heavy load, variable working conditions, and elevated temperature. Generated faults will lead to unexpected downtime, enormous economic loss, and sometimes security incidents. Machine fault diagnosis, which is designed to detect faults before failure happens, is one of the most essential systems in a wide range of rotating machinery. However, in practical industrial situations, the acquired data are significantly affected by the operating conditions, environment, and data acquisition devices, which may lead to unreliable diagnostic results [1,2]. Therefore, how to perform diagnosis efficiently and precisely is a challenging and worthwhile problem.

Traditional intelligent diagnosis methods mainly consist of three main stages: data collection, artificial feature extraction, and health state recognition [2,3]. However, artificial feature extraction greatly relies on the engineers’ specialized prior knowledge, and it is difficult to manually design a set of features that are applicable for all conditions. Furthermore, it is difficult for the generalization performance of traditional diagnosis models to bridge the relationship between massive data and health states [2,3,4].

Deep learning (DL) methods provide effective solutions to overcome the above limitations. Deep learning methods are able to automatically select discriminative features that are useful for making accurate predictions and learning nonlinear representation of the raw signal to a higher level of abstraction according to the training data [5]. Different kinds of deep learning techniques, such as deep belief networks (DBN), the stacked auto-encoder (AE), and the convolutional neural network (CNN) have been applied in fault diagnosis [2,6,7]. Among them, fault diagnosis approaches using convolutional network have developed most rapidly, and a lot of research work has been published [6]. Janssens et al. [8] utilized the discrete Fourier transform to process the accelerometer signals and presented a simple convolutional network for bearing condition recognition. Cao et al. [9] proposed a transfer learning approach based on AlexNet, with time-domain images as input without special processing, for gearbox fault diagnosis. Xin et al. [10] used short-time Fourier transform (STFT) to calculate time-frequency features and proposed a fault diagnosis method using AE to extract time-frequency features and using CNN to filter the useful features and obtain the faults classification. Ma et al. [11] proposed a fault diagnosis method of planetary gearbox under nonstationary running conditions using deep residual network with demodulated time-frequency features. Jin et al. [12] introduced attention modules into the residual network, with time-frequency graphs from STFT as the input. An active learning approach is introduced to achieve the same results using few compound faults samples. Shao et al. [3] presented a transfer learning approach for fault diagnosis. In this approach, the vibration data were converted to the 2D time—frequency images by wavelet transform (WT), and the images were used to fine-tune the pre-trained VGG-16 model. Guo et al. [13] employed the continuous WT to decompose vibration signals into scalogram according to the rotating speed. Then, a Pythagorean spatial pyramid pooling-based convolutional network was presented for bearing fault diagnosis. Zhao et al. [14] developed a variant of deep residual networks for planetary gearbox fault diagnosis under serious noise environment, and the input of the model is dynamically weighted wavelet coefficients. Furthermore, they [15] proposed the multiple wavelet coefficients fusion-based deep residual network for planetary gearbox fault diagnosis, which aimed to learn more easily distinguished features from the input data. Cao et al. [16] presented a CNN-based tool wear state recognition technique using machine spindle vibration signals. The proposed technique converted signals into graphs as the input of CNN, employing derived wavelet frames (DWFs). Theodoropoulos et al. [17] created the dataset through the signals from the sensors located on a real bulk carrier, and they proposed the use of CNN on colored contour images extracted from the dataset to recognize patterns that indicate early signs of defective behavior.

With the rapid development of internet technologies and the internet of things, the volume of collected data that is dramatically gathered is larger than ever before. However, lots of factors may lead to the poor-quality data, in practical industry, which has a significant difference from the experimental or simulated scenario [2,6]. Thus, more attention should be paid to the real-world industrial environment [18]. The main problem in the industrial application of vibration diagnostics is the masking of an informative signal by interfering signals [19]. The interfering signals can mainly be divided into high-frequency noise and low-frequency trend items [20,21,22]. A number of methods are presented for the separation of informative signals from background signals [20,21,22,23,24]. However, due to the difficulty of obtaining all the key information, it is difficult to propose a general and accurate separation signal algorithm for all conditions. Therefore, it is important to improve the robustness of the model for interfering signals.

In the current field of intelligent fault diagnosis, different researchers recommended using different input types and setting different hyper-parameters. It is crucial to evaluate and compare different DL-based intelligent diagnosis algorithms. Zhao et al. [25] made a great contribution to this issue. However, there are still many aspects to be further explored, such as deeper neural networks, input image size, and the performance of different input types under interfering signals. Almost all DL-based models mentioned in the above literature use one type of input, which is difficult to comprehensively reflect the fault feature information especially when the practical signal is mixed with interference information [26,27].

To address the problems above, in this paper, we evaluate different input types, both theoretically and experimentally, and propose multi-stream convolutional neural networks for rotating machinery fault diagnosis. The main contributions of this paper can be summarized as follows.

(1) Based on three public datasets, we conduct comprehensive experiments on four input types: time domain input, frequency domain input, STFT-based time–frequency input, and WT-based time–frequency input, with networks of three depths, two input sizes, and three types of interfering signals. Through the experiments, the suitable neural network depths and image sizes for these four input types are further obtained.
(2) Through theoretical analysis as well as analysis of experimental results, we study the difference in characteristics between the four input types, including the carried information, robustness to noise and trend items, learning difficulty for CNN models, etc. It preliminarily demonstrates the complementarity of information between different input types.
(3) We design a series of fusion models and conduct experiments to investigate where and how to fuse the three networks. Based on this, we proposed the final multi-stream convolutional neural network, which performs well under different environments, without any data pre-cleaning.
(4) We try to explore the inner mechanism of the proposed model by visualizing the learned feature maps. The feature distributions learned by different streams are different, which further demonstrates the complementarity of information. The fusion layers can fuse the input features well and help improve the classification ability of the network.

## 2. Input Types Definition and Discussion

### 2.1. Input Types Definition

#### 2.1.1. Time-Domain Input

**Figure 1:**
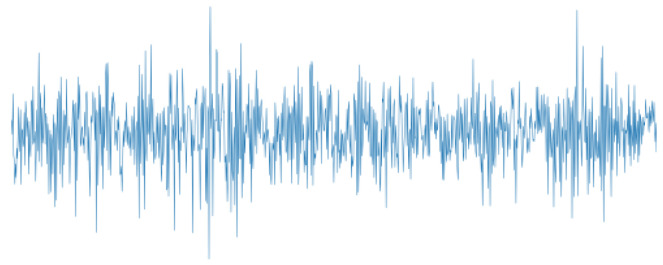
Image of time-domain input (TD).

Time-domain input is a preprocessing-free approach to transform the vibration signals to two-dimensional (2D) images. It just needs to connect the adjacent data points in chronological sequence to generate a polyline. Figure 1 illustrates an example of such a polyline generated by Matplotlib-Python. In the following, time-domain input is represented as TD.

#### 2.1.2. Frequency-Domain Input

FFT (Fast Fourier Transform) is applied to transform the time-domain vibration signals into frequency-domain ones, which is given by the following equation:

(1) $${\mathrm{FFT}}_{x}\left(f\right)=\int_{-\infty }^{+\infty }x\left(t\right)e^{-j2\pi ft}d_{t}$$

**Figure 2:**
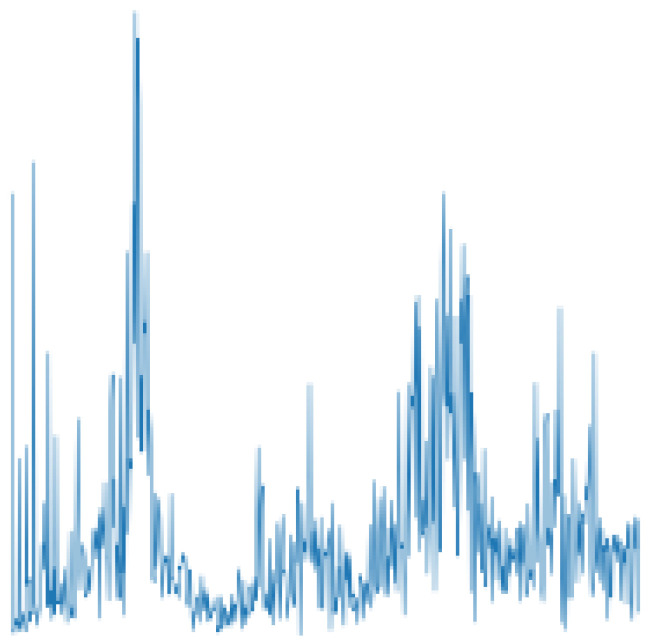
Image of FFT-based frequency-domain input (FD).

After this operation, we take the first half of the result and use it to generate the polyline, the same way as for TD. In the next article, we will use FD to denote the FFT-based frequency-domain input, and Figure 2 shows an example of FD.

#### 2.1.3. STFT Based Time–Frequency Domain Input

**Figure 3:**
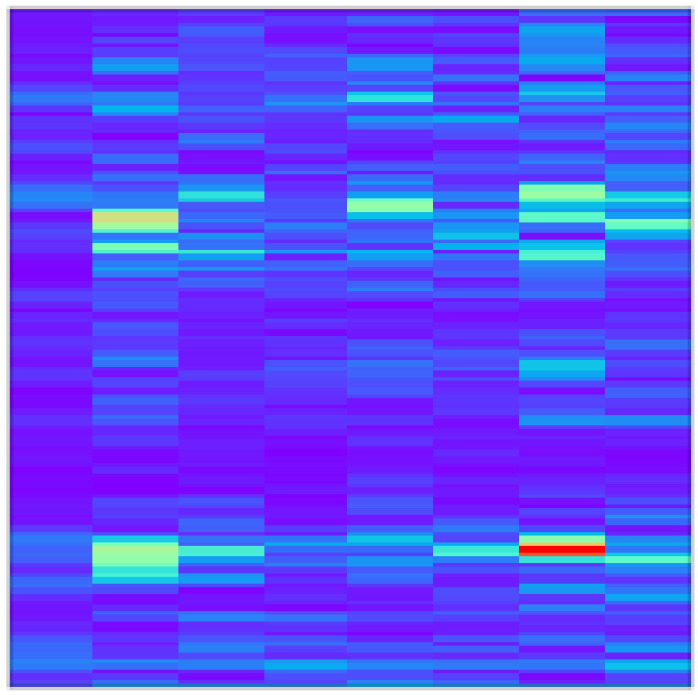
Image of STFT-based time–frequency domain input (STFT-TFD).

STFT (Short-time Fourier transform) adds the time variable to the Fourier transform through the sliding window on signals at the same stride. Moving the window and applying the Fourier transform to each segment leads to STFT:

(2) $${\mathrm{STFT}}_{x}\left(f,t\right)=\int_{-\infty }^{+\infty }x\left(\tau \right)g\left(\tau -t\right)e^{-j2\pi ft}d_{\tau }$$

where $x\left(\tau \right)$ is the monitoring data, and $g\left(\tau -t\right)$ is the window function. The observed signal through the window is $x\left(\tau \right)g\left(\tau -t\right)$. The Hanning window is used and the window length is 256 to balance time and frequency resolution. Here, the pseudo-color map is used to visually display the time–frequency characteristics. A visual representation example of the STFT-based time–frequency input (STFT-TFD) is shown in Figure 3 where the *x-* and *y*-axis are time and frequency, respectively, and the color scale of the image indicates the amplitude of the frequency.

#### 2.1.4. WT-Based Time–Frequency Domain Input

The WT (Wavelet Transform) is also widely used in fault diagnosis tasks. WTs are linear time–frequency representations with a wavelet. The WT of a signal, which is energy limited $x\left(t\right)\in L^{2}\left(R\right)$, can be set as

(3) $${\mathrm{WT}}_{x}\left(s,t\right)=\frac{1}{\sqrt{s}}\int_{-\infty }^{+\infty }x\left(\tau \right)\psi \left(\frac{\tau }{s}-t\right)d_{\tau }$$

where *s* is scale parameter; *t* is time parameter; and ψ is analyzing wavelet.

**Figure 4:**
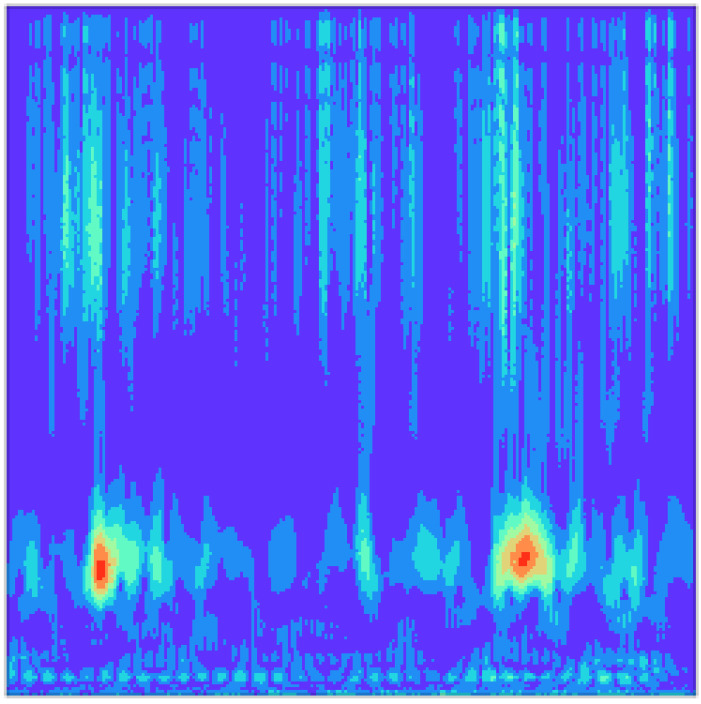
Image of WT-based time–frequency domain input (WT-TFD).

There is still no general consensus as to which wavelet can offer an optimal performance for fault diagnosis [15]. In this paper, the Morlet wavelet is chosen because of its similarity to the impulse component of symptomatic faults of many mechanical systems [28]. The pseudo-color contour map is applied to visually display the WT-based time–frequency input (WT-TFD), as Figure 4 illustrates.

### 2.2. Input Types Discussion

Raw sensory data are naturally time-series signals. When faults occur in rotating machinery, the time-domain signal usually changes. Its vibration amplitude, energy, and distribution are normally different for different health states [26,29]. In traditional intelligent diagnosis, the extraction of time-domain features is essential [26,27,29,30,31]. The time-domain features can be divided into the dimensional ones and the dimensionless ones. The former includes mean, standard deviation, root amplitude, etc., which are affected by the speed and the load of machines. The later contains shape indicator, kurtosis, crest indicator, etc., which are robust to the operation conditions [2].

Rotating machinery has a periodical impulse. Compared with the time-domain signal, the frequency spectrum can better highlight periodic information. In varying health states, characteristics of the frequency spectrum will be different, such as the vibration energy in the frequency domain, dispersion of frequency spectrum, position of main frequencies, and convergence of the spectrum power [26,30]. Its frequency resolution is extremely high, but it loses all temporal information. So, the frequency spectrum is commonly used for stationary signals [32]. In addition, its geometric structure is relatively simple, making it easy for CNN to extract features.

The time–frequency domain input represents a signal in both the time and frequency domains simultaneously. Some machines often switch between different running states, such as wind power generators and gas turbines, and the corresponding operation parameters may vary continuously in these transient processes, thus resulting in the nonstationary signal [13]. In addition, time–frequency representations have enormous advantages for identifying nonstationary signals [14,33]. Inputs in the time–frequency domain are pretty widely used in intelligent fault diagnosis, and their application is almost the most frequent in studies based on CNN [10,11,12,13,14,15,16].

This section provides a preliminary analysis of the characteristics and differences of the different input types. In Section 3, further analysis will be conducted through experiments.

## 3. CNN-Based Fault Diagnosis Evaluations

This section aims to explore the characteristics of different input types as well as suitable graph sizes and network depths. For the four input types, we conduct a series of evaluations with two input sizes, three depths of neural network, and three types of interference.

In this paper, each sample contains 1024 points, and the total number of samples can be obtained as follows:

(4) $$N=\mathrm{floor}\left(\frac{L}{1024}\right)$$

After generating samples, we randomly take 50% of the total samples as the training set, 20% of the total samples as the evaluating set, and 30% of the total samples as the testing set. The division ratio of the training set, validation set, and test set within each category is in the same proportion as the overall. During model training, we use Stochastic Gradient Descent (SGD) as the optimizer, Cross-Entropy Loss as the loss function, and test accuracy as the fault diagnosis accuracy. As a commonly used evaluation index for multi-classification tasks, the test accuracy can intuitively reflect the prediction situation, and it is used as the fault diagnosis accuracy.

### 3.1. Datasets

#### 3.1.1. CWRU Bearing Datasets

Case Western Reserve University (CWRU) datasets were provided by the Case Western Reserve University Bearing Data Center [34]. Single-point bearing defects were simulated by the electro-discharge machining. The accelerometers were attached to the drive end and fan end of the motor housing to collect vibration at 12 kHz or 48 kHz. This dataset was constructed under four motor loads, including 0 hp/1797 rpm, 1 hp/1772 rpm, 2 hp/1750 rpm, and 3 hp/1730 rpm. The data collected from the drive end at 12 kHz are used in this paper. It is classified into 15 health states, containing one health state and 14 fault states, as shown in Table 1.

#### 3.1.2. UoC Gear Fault Datasets

University of Connecticut (UoC) gear fault datasets were provided by the University of Connecticut [9,35]. The vibration was collected at 20 kHz. In this dataset, nine different gear conditions were introduced to the pinions on the input shaft, including healthy condition, missing tooth, root crack, spalling, and chipping tip, with five different levels of severity. In the original dataset, there are 104 samples per class and each sample has 3600 points. In this paper, each sample contains 1024 points; thus, the number of samples per class is expanded to 312.

#### 3.1.3. SEU Gearbox Datasets

Southeast University (SEU) gearbox datasets were provided by Southeast University [3,36]. This dataset contained two subdatasets, including a bearing dataset and a gear dataset. There were two kinds of working conditions with the rotating speed–load configuration set to be 20 Hz–0 V and 30 Hz–2 V. In this paper, the used dataset is the mixture combined with gear and bearing subdatasets including four kinds of gear failure, four kinds of bearing failure, and one health state under two working loads. There are nine health states, and each health state contains 2000 samples, as shown in Table 2. In addition, the volume of the SEU dataset is much larger than that in the other two datasets. Therefore, in this paper, 22%, 50%, and 100% of the SEU dataset are used as three independent datasets to investigate the effect of data volume on the fault diagnosis results.

### 3.2. CNN Models

CNN is a specialized kind of neural network that uses three basic ideas: local receptive fields, shared weights, and pooling. Its special architecture makes convolutional networks fast to train using fewer parameters compared with fully connected neural networks. In this section, the evaluations are based on VGG [37] and ResNet [38] unfolding. These two kinds of neural networks are classic and far-reaching, and many methods in the CNN field are built on their foundations.

The VGG architecture is proposed by the Visual Geometry Group at Oxford University. Their main contribution is a thorough evaluation of networks of increasing depth using an architecture with very small (3 × 3) convolution filters, which shows that a significant improvement on the prior-art configurations can be achieved by pushing the depth to 16–19 weight layers [37]. In this paper, VGG-16 that contains 13 convolutional layers and three fully concatenated layers is used as the shallower convolutional neural network.

**Figure 5:**
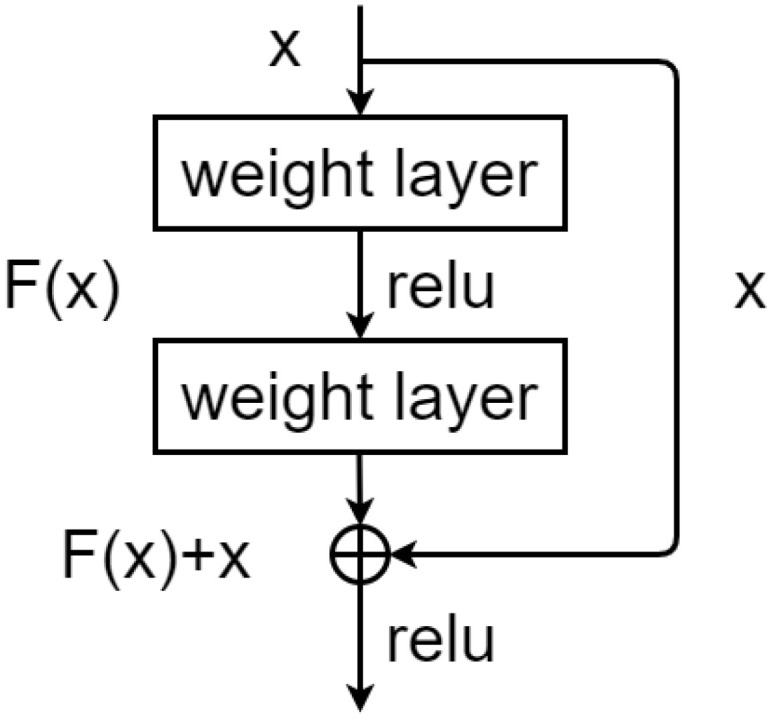
Architecture of the residual block.

Deep Residual Network (ResNet) is a groundbreaking work in the computer vision and deep learning. The residual learning framework was presented to overcome the degradation problem of deep networks. Residual block is the basic component of ResNet. It introduces the identity shortcut connection that skips one or more convolutional layers, as shown in Figure 5. With *x* as an input map of a residual block, the output *y* of the residual block is obtained as follows:

(5) $$y=\sigma \left(F\left(x,\left\{\omega_{i}\right\}\right)+x\right)$$

where the function $F\left(x,\left\{\omega_{i}\right\}\right)$ represents the residual mapping to be learned and σ is the activation function ReLU.

Using ResNet18 and ResNet34 directly in fault diagnosis evaluations, the results obtained are sometimes degraded compared to VGG16, especially when TD is used as input. After experimental verification, the reason is that ResNet reduces the resolution of the image in the initial stage of convolution. Therefore, we adjust ResNet18 and ResNet34 in structure, as shown in Table 3. In this paper, the adjusted ResNet18 and ResNet34 are used as medium-depth and deep neural networks, respectively, and we call them ResNet18’ and ResNet34’.

### 3.3. Evaluations without Interfering Signals

We first discuss the experimental results of the four input types in different neural networks and input sizes, without interference. CWRU, UoC, 22%SEU, 50%SEU, and 100%SEU with no additional interfering signals are used as the five datasets. The two different input image sizes are 256 × 256 and 128 × 128. The three CNN models are VGG16, ResNet18’, and ResNet34’. The test accuracies of evaluations are divided into five groups by dataset as shown in Table 4, Table 5, Table 6, Table 7 and Table 8.

Through the analysis of Table 4, Table 5, Table 6, Table 7 and Table 8, we can get the following results. TD performs better at high resolution and deep network. In most cases, FD performs best on VGG16, and STFT-TFD and WT-TFD perform best on ResNet18’. In the following interference experiments, the networks used for TD, FD, STFT-TFD, and WT-TFD are ResNet34’, VGG16, ResNet18’, and ResNet18’, respectively. Since the performance of FD, STFT-TFD, and WT-TFD did not vary much under different input sizes in this round of experiments, further experiments on input sizes are needed.

### 3.4. Evaluations with Interfering Signals

We design three configurations of experiments to further explore the performance of the four input types under interfering signals and their sensitivity to image resolution. Generally, the interfering signals can be divided into high-frequency noise and low-frequency trend items. We simulated three types of interfering signals: noise, trend items, and noise plus trend items. The three types of interfering signals are introduced to the three configurations of experiments, respectively.

Verstraete et al. [4] and Zhang et al. [18] only used white Gaussian noise to simulate the noise environment. However, white Gaussian noise has limited influence on the distribution of the main frequencies. In order to better simulate the actual industrial environment, we randomly add the real vibration (RV) from other equipment or white Gaussian noise (WGN) to the datasets. Random numbers α and ρ are first generated, and the simulated noise is given by following equation:

(6) $$\mathrm{noise}=\left\{\begin{array}{cc} \rho \cdot \mathrm{WGN} & \alpha <0.5\\ \rho \cdot \left(\theta \cdot \mathrm{RV}\right) & \alpha \geq 0.5 \end{array}\right.$$

where $\alpha \in \left[0,1\right]$ and $\rho \in \left[0,1\right]$ are two independent random numbers, and θ is the dimensionless factor used to scale the RV to the same power as WGN. For the CWRU, the RV comes from the vibration of the SEU’s motor, and for the SEU and UoC, the RV comes from the vibration of the CWRU’s motor base, at 12 kHz. In this paper, the maximum powers of the noise in each dataset are certain, which can be quantified by the SNR of the noise to the vibration in a healthy state under low load. The definition of SNR is shown as follows:

(7) $${\mathrm{SNR}}_{db}=10\log_{10}\left(\frac{P_{s}}{P_{n}}\right)$$

where $P_{s}$ is the power of the signal in the health state under low load and $P_{n}$ is the power of noise. The minimum SNR values of CWRU, UoC, and SEU are −15, −4.5, and −1.5, respectively.

There are various reasons for trend items, which may be the influence of the signal acquisition system by temperature, humidity, electromagnetic field, etc., or the basic motion of the machine [33,39,40,41]. To simulate the diversity of trend items, we chose a series of basis functions. The set of bases is as follow:

(8) $$F=\left\{a_{1}\sqrt{x},a_{2}x,a_{3}x^{2},a_{4}\sin\left(b_{1}x\right),a_{5}sin\left(b_{2}x^{2}\right)\right\}$$

where $a_{1}$, $a_{2}$, $a_{3}$, $a_{4}$, $a_{5}$, $b_{1}$, and $b_{2}$ are the independent random numbers to adjust the bases and add randomness to the trend items. With the value range of *x* is {0, 1/1024, 2/1024, …, 1023/1024}, the value ranges of the parameters of the five datasets are shown in Table 9.

Random numbers γ and β are first generated, and the trend items is given by following equation:

(9) $$\mathrm{tendency}=\left\{\begin{array}{cc} 0 & \beta <0.5\\ \gamma \cdot f & 0.5\leq \beta <0.8\\ \gamma \cdot f+\gamma^{\prime }\cdot f^{\prime } & \beta \geq 0.8 \end{array}\right.$$

where *f* and $f^{\prime }$ are the randomly selected basis functions from the set *F*, $\beta \in \left[0,1\right]$ and $\gamma \in \left\{-1,1\right\}$ are independent random numbers.

Evaluations with interfering signals are conducted through introducing the simulated interference to the datasets before training. The results are shown in Table 10, Table 11 and Table 12.

Based on these three configurations of experiments, we can make the following analysis and summary:

(1) TD is significantly affected by input size and data size, and it always performs better at higher resolutions and larger data sizes. It can be shown from Table 10 that TD performs the worst with noise compared to the other three input types containing frequency domain information, which means its poor robustness to noise. By comparing the performance of TD in different situations, it can be seen that TD is almost unaffected by the trend items. Surprisingly, in Table 12, the performance of TD on 50% SEU and 100% SEU is the best among the four input types, indicating that TD contains rich health information but is difficult to train and hard to fit.
(2) As can be seen from Table 11 and Table 12, FD requires higher resolution to achieve better prediction accuracy when the interfering signal contains trend items, but it is less affected by image resolution than TD. In most cases, FD performs best with interference containing only noise, indicating its great robustness to noise. However, FD is less robust to trend items relative to TD, which should be due to the fact that the frequencies of trend items may mask the main frequencies of high frequencies.
(3) STFT-TFD is insensitive to resolution and often performs better at lower resolution. STFT-TFD has excellent robustness to trend items, almost unaffected, and its robustness to noise is also good.
(4) WT-TFD is insensitive to resolution, and its performance is greatly affected by data scale. WT-TFD is more robust to noise than TD, but it performs poorly under trend items.

We obtain the performance of four input types under different input sizes, different depths of CNN models, and different types of interfering signals, based on Table 4, Table 5, Table 6, Table 7 and Table 8 and Table 10, Table 11 and Table 12. Through the analysis of the experimental results of this section and the theoretical analysis of Section 2, it can be concluded that different input types differ in many characteristics, which preliminarily proves the information complementarity.

## 4. Proposed Method

The proposed method is aim to automatically extract and fuse time domain, frequency domain, and time–frequency domain features, fully exploit the health information contained in the vibration signal, improve the robustness of the model to interfering signals, and obtain higher fault diagnosis accuracy.

We choose three input types for three streams and investigate where and how to perform information fusion. The final multi-stream convolutional neural network is proposed in Section 4.2 and compared with the single-input models in Section 3 and other advanced fault diagnosis methods. We visualize the proposed model and further demonstrate the complementarity of information between different input types.

### 4.1. Where and How to Fuse the Streams

The proposed method contains three streams, time domain stream (TD-stream), frequency domain stream (FD-stream), and time–frequency domain stream (TFD-stream). For two kinds of time–frequency domain inputs, STFT-TFD is more robust than WT-TFD to both trend items and noise. So, STFT-TFD is chosen as the input for the time–frequency domain stream. Therefore, the inputs of the three streams are TD, FD, and STFT-TFD. In Section 3, we explore suitable input sizes for different input types. TD and FD require high resolution, while STFT-TFD is insensitive to resolution. Therefore, the map sizes of the three input types are 256 × 256, 256 × 256, and 128 × 128, respectively. The training difficulty of the three input types is different for CNN. According to the conclusions in Section 3.1, the convolutional networks designed to extract the features of TD, FT, and STFT-TFD are based on ResNet34’, VGG16, and ResNet18’.

When performing multi-stream information fusion, there are two main problems: where to and how to fuse the three networks. In the following, we will study these two problems through a sets of experiments.

**Figure 6:**
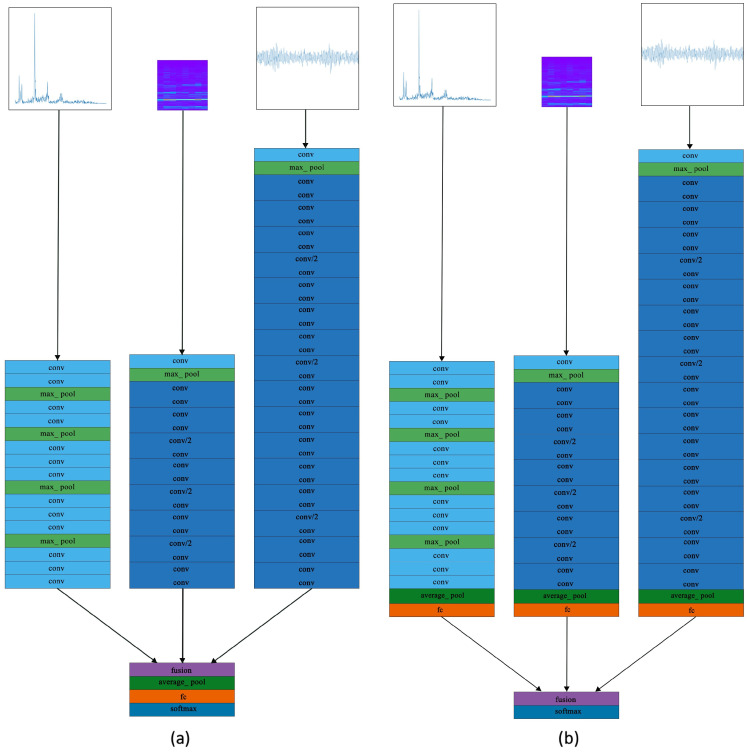
Two examples of multi-stream networks: (**a**) the structure fusing information after Conv and (**b**) the structure fusing information after Fc.

Since the three input types do not have a strict pixel correspondence, fusion is not suitable to be performed at an early stage. The fusion layer is injected after the last convolutional layer (Conv), average pooling layer (Ap), fully connected layer (Fc), and softmax layer. Figure 6 shows the network structures fusing information after Conv and Fc, and the other two structures are similar.

A fusion function applied to fuse the feature maps into one output feature map can be defined as follows:

(10) $$y=f\left(x^{a},x^{b},x^{c},\cdots \right)$$

For simplicity, we define the fusion layer with two three-dimensional input feature maps. It is easy to be extended to multiple inputs in the same or different dimensions. In this case, the operation of a fusion layer can be defined as:

(11) $$y=f\left(x^{a},x^{b}\right)$$

where $x^{a}\in R^{H\times W\times D}$ and $x^{b}\in R^{H^{\prime }\times W^{\prime }\times D^{\prime }}$ are the inputs, $y\in R^{H^{\prime \prime }\times W^{\prime \prime }\times D^{\prime \prime }}$ is the output, and f(·) represents the mapping relationship between inputs and output. *W*, *H*, and *D* are the width, height, and number of channels of the respective feature maps, and we assume that $H=H^{\prime }=H^{\prime \prime }$, $W=W^{\prime }=W^{\prime \prime }$, and $D=D^{\prime }$. When the inputs of the fusion layer have different map resolutions, we downsample the high-resolution maps, which is achieved through stride-2 3 × 3 convolutions, to solve this problem. The mapping relationship f(·) can be implemented in several ways, and three public and classical ways are used in this paper.

Sum fusion computes the sum of the two feature maps at the same spatial locations *i*, *j*, and feature channels *d*:

(12) $$y_{i,j,d}^{sum}=x_{i,j,d}^{a}+x_{i,j,d}^{b}$$

where 1≤i≤H, 1≤j≤W, 1≤d≤D and $x^{a},x^{b},y^{sum}\in R^{H\times W\times D}$.

Cat fusion stacks the two feature maps at the same spatial locations *i*, *j* across the feature channels *d*:

(13) $$\left\{\begin{array}{c} y_{i,j,d}^{cat}=x_{i,j,d}^{a}\\ y_{i,j,D+d}^{cat}=x_{i,j,d}^{b} \end{array}\right.1\leq d\leq D$$

where $y^{cat}\in R^{H\times W\times 2D}$.

Conv fusion first stacks the two feature maps at the same spatial locations *i*, *j* across the feature channels *d* as above (13) and subsequently convolves the stacked data with a bank of filters $\omega \in R^{3\times 3\times 2D\times D}$ and biases $b\in R^{D}$:

(14) $$y^{conv}=\sigma \left(y^{cat}*\omega +b\right)$$

where σ is the activation function ReLU, ∗ is the convolution operator, and $y^{conv}\in R^{H\times W\times D}$. Actually, Conv fusion is equivalent to performing convolution after Cat fusion.

We conduct multi-stream information fusion evaluations based on the datasets CWRU, UoC, and 22%SEU with noise plus trend items, and the results are shown in Table 13.

In the obtained experimental results, all the test accuracies are higher than the test accuracies shown in Table 13. As for the fusion method, Sum fusion performs the best in most cases, and the calculation amount added by Sum fusion to the network is almost negligible. Therefore, Sum fusion is recommended as the method of information fusion. The fusion after Softmax performs stable and excellent; in addition, the fusion after Ap also achieves good results in CWRU and UoC.

### 4.2. Multi-Stream Convolutional Neural Network

**Figure 7:**
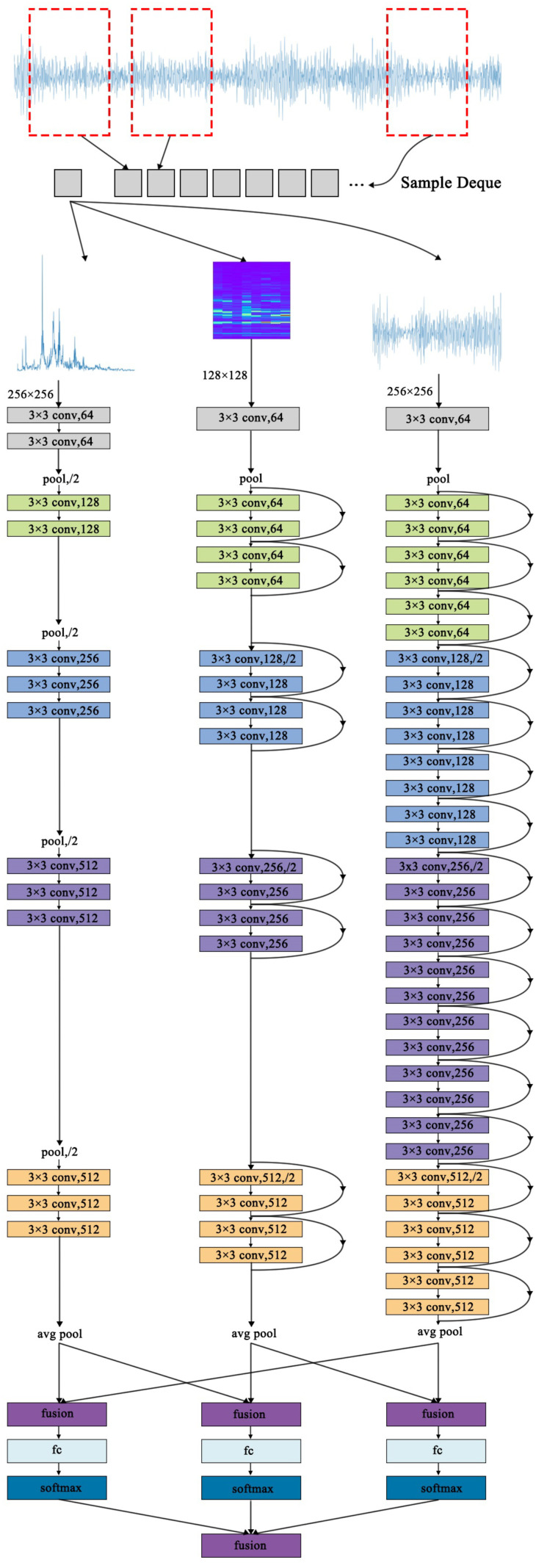
Structure of the final proposed network.

The structure of the final proposed multi-stream convolutional neural network is shown in Figure 7. The vibration data are first segmented, and every 1024 points is taken as a sample. One sample generates TD, FD, and STFT-TFD simultaneously as the input of the three convolutional streams. In order to fully fuse the health information contained in the three streams, we fuse the three networks twice, with the fusion positions after Ap and Softmax, respectively. All the fusion layers in the proposed structure are implemented by Sum fusion.

In order to test the improvement of information fusion on diagnostic accuracy and the robustness of the proposed model to interference, we first test the proposed model on five datasets with noise plus trend items. On the same datasets with the same noise and trend items, the proposed model is compared with the single-input fault diagnosis models in Section 3 and the methods from sources [25,41]. We denote the best performance, in Section 3, with different input types and input sizes as the baseline. The one-dimensional (1D) residual network proposed by [25] is chosen to be the comparison object, and its input is the 1D vibration signal without any process. Hasan et al. [41] proposed a multidomain input type, where the three RGB channels of the input image are the time domain, the frequency domain, and the inclusive grayscale image, respectively. It is also compared with our proposed method using the same training set proportions and evaluation criteria as this experiment. The test accuracies among these methods are listed in Table 14. The proposed method yields an improvement of 2.33–5.3% compared with the baselines. Compared to the other two methods, the proposed method not only obtains higher test accuracy but also shows excellent generalization to different datasets.

**Figure 8:**
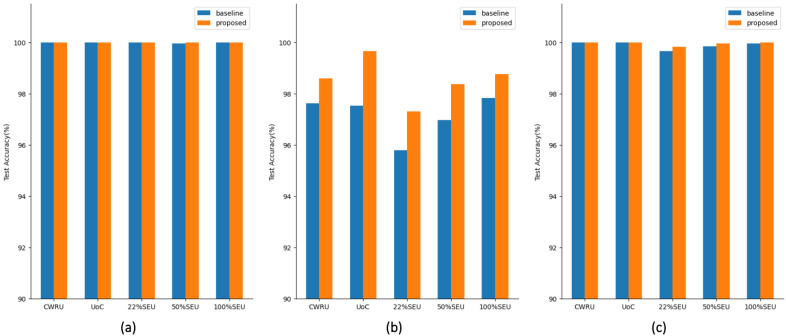
Performance comparisons: (**a**) tests on original datasets, (**b**) tests on datasets with noise, and (**c**) tests on datasets with trend items.

We also test the performance of the proposed structure on the original datasets and datasets with noise or trend items. The comparisons are shown in Figure 8. It can also be seen that the fault diagnosis accuracy is improved in all three cases.

According to Table 14 and Figure 8, our model has a larger improvement in test accuracy when the amount of data is lower. In this paper, the training set accounts for 50%, while the proportion of the training set can reach 80% in practical applications. With the help of cross-validation, transfer learning, data enhancement, and other methods, the dependence on the amount of data can be further reduced. As a conservative estimate, for the proposed model, we recommend that the number of datasets per class be not at least 190 samples (194,560 sampling points).

**Figure 9:**
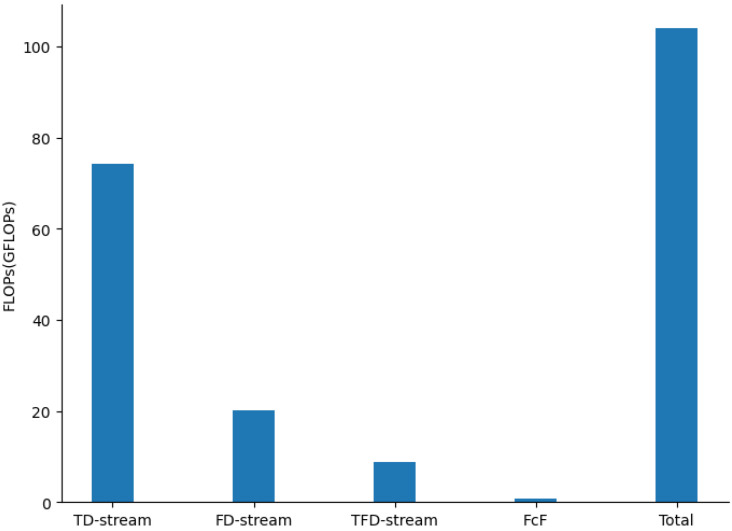
FLOPs comparisons.

The number of total parameters of the proposed model is 49.54 M, the floating-point operations (FLOPs) [42] is 104.01 GFLOPs, and the memory usage is 1058.00 MB. We also separately compute FLOPs for the three convolutional streams and fully connected and fusion layers (FcF) by comparing them with the total FLOPs, as shown in Figure 9. It can be seen that the computational cost of the proposed mainly comes from TD-stream, and the structure of TD-stream has the potential for optimization.

**Figure 10:**
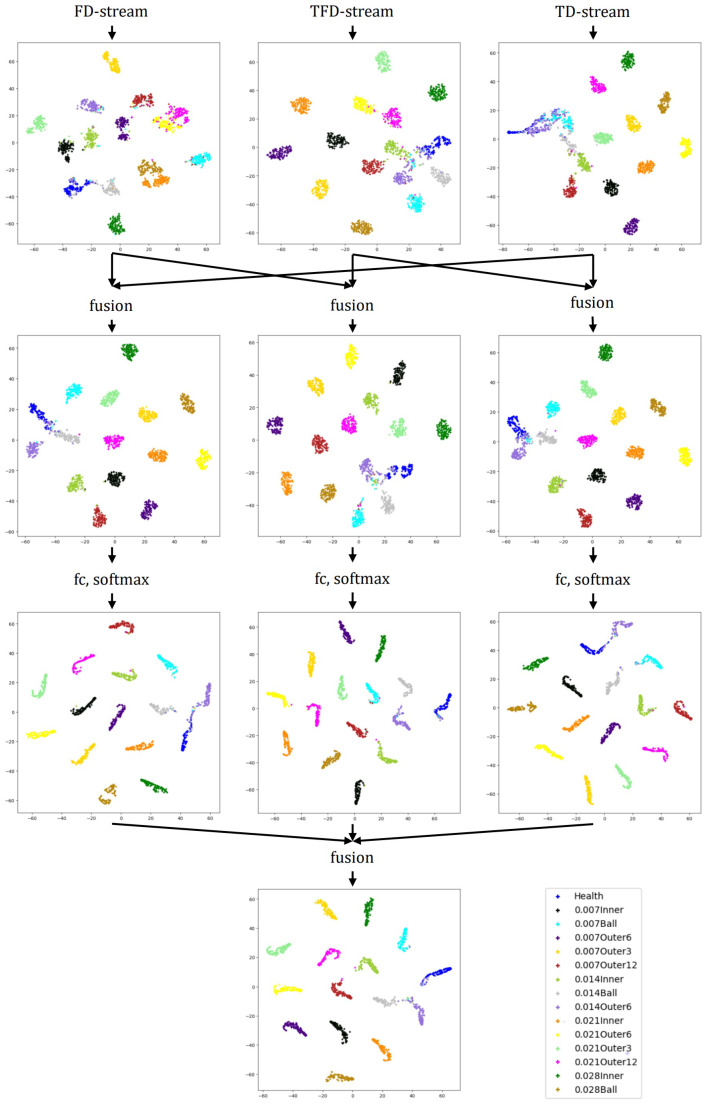
Feature maps visualization for CWRU with noise and trend items.

**Figure 11:**
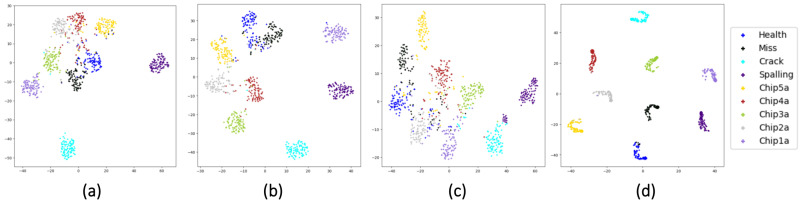
Feature maps visualization for UoC with noise and trend items: (**a**) visualization of FD-stream before the first fusion, (**b**) visualization of TFD-stream before the first fusion, (**c**) visualization of TD-stream before the first fusion, and (**d**) visualization after the second fusion.

**Figure 12:**
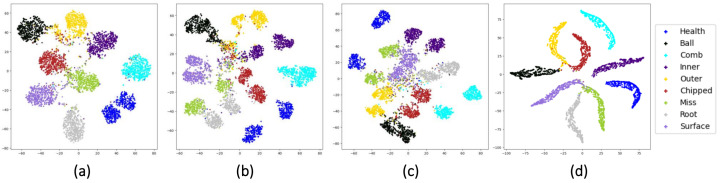
Feature maps visualization for SEU with noise and trend items: (**a**) visualization of FD-stream before the first fusion, (**b**) visualization of TFD-stream before the first fusion, (**c**) visualization of TD-stream before the first fusion, and (**d**) visualization after the second fusion.

To get a further sense of the feature learning ability of different stream networks and the function played by the fusion layers, we use t-SNE [43] to visualize the feature maps before and after two times of fusion. The t-SNE is a nonlinear dimensionality algorithm, which is highly suitable for visualizing high-dimensional data in 2D or three-dimensional (3D) feature space. The complete visualization for CWRU with noise and trend items is shown in Figure 10. For the datasets UoC and SEU with noise and trend items, we only show the visualization of feature maps before the first fusion and after the second fusion in Figure 11 and Figure 12.

As shown in Figure 9, the learned feature distributions are different for the three streams. For example, the learned features of the TD-stream are hard to distinguish between Health, 0.007Ball, and 0.014Outer6. The reason for it may be that the vibration signals in the three types of healthy state have small amplitudes, which are easily masked by noise and indistinguishable from TD. The FD-stream distinguishes these three classes well, but its aggregation of a single health state is poor. The TFD-stream has slightly weak classification ability for health, 0.14Outer6 and 0.014Inner. From Figure 10, it is also shown that fusion layers are of great help to overcome the shortcomings of a single stream for fault diagnosis and improve the aggregation of identically labeled data and the separation of disparately labeled data. In Figure 11 and Figure 12, we can also claim that the distribution difference between the features learned by the convolutional networks of different streams is huge, and the final output layer obtains a good classification result after two times of fusion.

## 5. Conclusions

In this paper, we analyze the differences of the four input types in many characteristics through theoretical analysis and extensive experiments. Based on this, we propose the new model named multi-stream convolutional neural network for rotating machinery fault diagnosis. The model takes time domain, frequency domain and time–frequency domain images as input and is able to fuse information from different inputs automatically. Our algorithm exhibits excellent robustness to noise and trend items and exceeds the state of the art. We demonstrate the information complementarity between different input types from multiple perspectives, including theoretical and experimental analysis, improvement in diagnostic accuracy, and visualization of the learned feature maps. However, the datasets used in this study do not contain nonstationary signals, and the model is tested on the gear and bearing datasets only. Therefore, we hope to further verify the performance of our method on unstable signals and different engineering areas datasets. Additionally, the proposed model is computationally intensive compared to many lightweight models. Future work will lighten the model, reducing the parameters and training time required for the model.

## Figures and Tables

**Table 1:** Detailed description of CWRU datasets.

Health State	Fault Position	Fault Diameter	Working Load	Sample Size
Health	-	-	0/1/2/3	110/110/110/110
0.007Inner	inner race	0.007	0/1/2/3	110/110/110/110
0.014Inner	inner race	0.014	0/1/2/3	110/110/110/110
0.021Inner	inner race	0.021	0/1/2/3	110/110/110/110
0.028Inner	inner race	0.028	0/1/2/3	110/110/110/110
0.007Ball	rolling element	0.007	0/1/2/3	110/110/110/110
0.014Ball	rolling element	0.014	0/1/2/3	110/110/110/110
0.021Ball	rolling element	0.021	0/1/2/3	110/110/110/110
0.028Ball	rolling element	0.028	0/1/2/3	110/110/110/110
0.007Outer6	outer race @6:00	0.007	0/1/2/3	110/110/110/110
0.007Outer3	outer race @3:00	0.007	0/1/2/3	110/110/110/110
0.007Outer12	outer race @12:00	0.007	0/1/2/3	110/110/110/110
0.014Outer6	outer race @6:00	0.014	0/1/2/3	110/110/110/110
0.021Outer6	outer race @6:00	0.021	0/1/2/3	110/110/110/110
0.021Outer3	outer race @3:00	0.021	0/1/2/3	110/110/110/110
0.021Outer12	outer race @12:00	0.021	0/1/2/3	110/110/110/110

**Table 2:** Detailed description of SEU datasets.

Health State	Working Load	Sample Size
Health	0/2	1000/1000
Chipped	0/2	1000/1000
Miss	0/2	1000/1000
Root	0/2	1000/1000
Surface	0/2	1000/1000
Ball	0/2	1000/1000
Inner	0/2	1000/1000
Outer	0/2	1000/1000
Combination	0/2	1000/1000

**Table 3:** Architectures of ResNet18’ and ResNet34’ with input size of 256 × 256.

Layer Name	Output Size	ResNet18’	ResNet34’
conv1	256×256	3×3,64,stride1	
conv2_x	256×256	3×3maxpool,stride1	
		$\left[\begin{array}{cc} 3\times 3, & 64\\ 3\times 3, & 64 \end{array}\right]$ ×2	$\left[\begin{array}{cc} 3\times 3, & 64\\ 3\times 3, & 64 \end{array}\right]\times 3$
conv3_x	128×128	$\left[\begin{array}{cc} 3\times 3,128 & \\ 3\times 3,128 \end{array}\right]\times 2$	$\left[\begin{array}{cc} 3\times 3,128 & \\ 3\times 3,128 \end{array}\right]\times 4$
conv4_x	64×64	$\left[\begin{array}{cc} 3\times 3,256 & \\ 3\times 3,256 \end{array}\right]\times 2$	$\left[\begin{array}{cc} 3\times 3,256 & \\ 3\times 3,256 \end{array}\right]\times 6$
conv5_x	32×32	$\left[\begin{array}{cc} 3\times 3,512 & \\ 3\times 3,512 \end{array}\right]\times 2$	$\left[\begin{array}{cc} 3\times 3,512 & \\ 3\times 3,512 \end{array}\right]\times 3$
	1×1	average pool, fc, softmax	

**Table 4:** Test accuracies of CWRU.

Networks	Input Sizes	TD	FD	STFT-TFD	WT-TFD
VGG16	256×256	99.70%	99.95%	99.92%	99.75%
	128×128	99.60%	99.95%	100.0%	99.75%
ResNet18’	256×256	99.80%	100.0%	100.0%	99.90%
	128×128	99.50%	99.95%	100.0%	99.85%
ResNet34’	256×256	99.85%	99.92%	100.0%	99.95%
	128×128	99.55%	99.95%	100.0%	99.95%

**Table 5:** Test accuracies of UoC.

Networks	Input Sizes	TD	FD	STFT-TFD	WT-TFD
VGG16	256×256	100.0%	100.0%	99.88%	100.0%
	128×128	99.88%	99.88%	100.0%	99.88%
ResNet18’	256×256	99.88%	100.0%	100.0%	100.0%
	128×128	99.65%	100.0%	100.0%	100.0%
ResNet34’	256×256	100.0%	100.0%	100.0%	100.0%
	128×128	100.0%	100.0%	100.0%	100.0%

**Table 6:** Test accuracies of 22%SEU.

Networks	Input Sizes	TD	FD	STFT-TFD	WT-TFD
VGG16	256×256	96.47%	100.0%	99.37%	97.48%
	128×128	95.96%	99.41%	98.91%	98.65%
ResNet18’	256×256	97.31%	99.66%	99.83%	99.16%
	128×128	96.04%	99.50%	99.37%	99.16%
ResNet34’	256×256	98.82%	99.83%	99.66%	99.16%
	128×128	96.30%	99.92%	99.33%	98.91%

**Table 7:** Test accuracies of 50%SEU.

Networks	Input Sizes	TD	FD	STFT-TFD	WT-TFD
VGG16	256×256	99.52%	99.82%	99.63%	99.59%
	128×128	97.67%	99.82%	99.48%	99.37%
ResNet18’	256×256	99.41%	99.82%	99.96%	99.67%
	128×128	97.74%	99.82%	99.74%	99.52%
ResNet34’	256×256	99.78%	99.96%	99.93%	99.63%
	128×128	98.30%	99.85%	99.59%	99.33%

**Table 8:** Test accuracies of 100%SEU.

Networks	Input Sizes	TD	FD	STFT-TFD	WT-TFD
VGG16	256×256	99.91%	99.98%	99.89%	99.78%
	128×128	99.20%	100.0%	99.82%	99.85%
ResNet18’	256×256	99.82%	99.91%	99.94%	99.93%
	128×128	99.04%	99.93%	99.82%	99.96%
ResNet34’	256×256	100.0%	99.96%	99.93%	99.96%
	128×128	99.22%	99.94%	99.91%	99.93%

**Table 9:** Value ranges of the parameters $a_{i}$ and $b_{j}$.

Parameters	CWRU	UoC	22%SEU	50%SEU	100%SEU
$a_{1}$	(1, 7)	(0.3, 0.7)	(0.01, 0.03)		
$a_{2}$	(1, 7)	(0.3, 0.7)	(0.01, 0.03)		
$a_{3}$	(1, 7)	(0.3, 0.7)	(0.01, 0.03)		
$a_{4}$	{1}	{0.1}	{0.01}		
$a_{5}$	{1}	{0.1}	{0.01}		
$b_{1}$	($10^{-3},10^{-2}$)	($10^{-3},10^{-2}$)	($10^{-3},10^{-2}$)		
$b_{2}$	($10^{-7},10^{-6}$)	($10^{-7},10^{-6}$)	($10^{-7},10^{-6}$)		

**Table 10:** Test accuracies of the evaluation with noise.

Datasets	Input Sizes	TD	FD	STFT-TFD	WT-TFD
CWRU	256×256	94.14%	97.63%	96.77%	96.11%
	128×128	92.88%	97.17%	97.07%	96.77%
UoC	256×256	94.92%	95.51%	96.81%	95.27%
	128×128	84.75%	93.97%	97.52%	96.81%
22%SEU	256×256	91.75%	95.37%	93.69%	93.18%
	128×128	82.66%	95.79%	94.11%	92.42%
50%SEU	256×256	94.85%	96.96%	94.26%	94.41%
	128×128	91.04%	96.48%	94.44%	92.93%
100%SEU	256×256	96.48%	97.82%	95.57%	96.13%
	128×128	93.35%	97.50%	96.17%	94.37%

**Table 11:** Test accuracies of the evaluation with trend items.

Datasets	Input Sizes	TD	FD	STFT-TFD	WT-TFD
CWRU	256×256	99.85%	99.75%	99.90%	89.65%
	128×128	99.44%	99.39%	100.0%	88.64%
UoC	256×256	100.0%	99.17%	100.0%	97.05%
	128×128	99.88%	99.05%	100.0%	96.69%
22%SEU	256×256	99.33%	98.65%	99.50%	96.89%
	128×128	96.59%	98.32%	99.66%	96.72%
50%SEU	256×256	99.85%	99.52%	99.83%	97.85%
	128×128	97.93%	99.26%	99.85%	97.63%
100%SEU	256×256	99.96%	99.80%	99.76%	98.70%
	128×128	99.24%	99.65%	99.87%	98.19%

**Table 12:** Test accuracies of the evaluation with noise plus trend items.

Datasets	Input Sizes	TD	FD	STFT-TFD	WT-TFD
CWRU	256×256	94.34%	96.01%	93.69%	85.15%
	128×128	93.49%	95.46%	95.86%	86.16%
UoC	256×256	94.56%	91.73%	93.74%	90.90%
	128×128	82.15%	90.07%	95.27%	89.36%
22%SEU	256×256	88.13%	90.66%	90.15%	88.90%
	128×128	81.57%	89.73%	91.67%	86.70%
50%SEU	256×256	94.78%	94.44%	92.67%	92.11%
	128×128	89.56%	93.52%	93.41%	89.67%
100%SEU	256×256	96.00%	95.67%	94.83%	93.78%
	128×128	92.74%	94.82%	94.98%	92.50%

**Table 13:** Performance comparison of different fusion strategies.

Datasets	Fusion Ways	Conv	Ap	Fc	Softmax
CWRU	sum	96.87%	97.53%	97.32%	97.68%
	cat	96.97%	97.12%	-	-
	conv	97.12%	97.67%	97.48%	-
UoC	sum	97.52%	97.40%	97.28%	97.99%
	cat	97.40%	96.57%	-	-
	conv	96.22%	96.45%	96.34%	-
22%SEU	sum	94.53%	94.70%	94.78%	95.37%
	cat	93.27%	93.35%	-	-
	conv	93.77%	94.44%	93.94%	-

**Table 14:** Test accuracies of different methods on datasets with noise plus trend items.

Methods	CWRU	UoC	22%SEU	50%SEU	100%SEU
[25]	97.22%	93.62%	75.34%	86.67%	90.61%
[41]	96.92%	91.49%	90.74%	93.59%	95.17%
baseline	96.01%	95.27%	91.67%	94.78%	96.00%
proposed	**98.33%**	**98.58%**	**96.97%**	**97.74%**	**98.59%**

## Data Availability

Not applicable.
